# Acrometastasis of Lung Adenocarcinoma to the Fibula: A Report of a Rare Case

**DOI:** 10.7759/cureus.75834

**Published:** 2024-12-16

**Authors:** Adahra P Beso, Neeharika Phukan, Barasha S Bharadwaj, Muktanjalee Deka, Lohit Kalita

**Affiliations:** 1 Oncopathology, State Cancer Institute, Gauhati Medical College and Hospital (GMCH), Guwahati, IND; 2 Oncosurgery, State Cancer Institute, Gauhati Medical College and Hospital (GMCH), Guwahati, IND

**Keywords:** acrometastasis, fibula, histopathology, immunohistochemistry, lung adenocarcinoma

## Abstract

Acrometastasis is an extremely rare diagnosis, invariably associated with poor prognosis. A 60-year-old female with complaints of cough and breathing difficulty also presented with pain and swelling in her left leg. Radiological investigations suggested a double primary in the lung and leg; histopathology and immunohistochemistry (IHC) confirmed the lesion in the leg to be metastatic from the lung primary. There should always be a high index of suspicion for acrometastasis in lung adenocarcinoma, as it is the most common malignancy to metastasize to the acral sites, followed by renal cell carcinoma, breast carcinoma, and colon carcinoma. The correct approach to tumors at several sites, in conjunction with history, radiology, and pathology, would aid in the timely and accurate diagnosis for proper patient management.

## Introduction

Bone metastasis is the most frequent form of malignant bone involvement, which predominantly affects the axial skeleton as compared to the appendicular skeleton [1]. Acrometastasis is a type of bone metastasis that is linked with a poor prognosis, reduced quality of life, and poor patient survival. It is an unusual occurrence typically linked to lung malignancies, especially non-small-cell lung cancer, particularly the adenocarcinoma subtype. Acrometastasis is defined as metastasis distal to the knee in the lower limb and distal to the elbow in the upper limb. In particular, acrometastasis in the hand constitutes about 0.07-0.2% of all metastases and about 0.1% of bony metastasis [2]. The patient usually presents with complaints of pain and swelling of the affected bony metastatic site, and it could also be the first presentation of an occult primary. It is extremely rare and most often misdiagnosed as either a metachronous or synchronous double primary or infection [3]. Carcinoma of the lung metastasizes to the liver, bone, adrenal gland, respiratory system, and nervous system with a dismal prognosis in bony metastases [4].

In this article, we present a case of lung adenocarcinoma with acral metastasis to the fibula. Although a rare scenario, this study attempts to raise awareness of acrometastasis and the myriad of clinical and diagnostic challenges associated with it.

## Case presentation

A 60-year-old female presented to the oncosurgery department with complaints of intermittent coughing and troubled breathing for the past year. She also reported experiencing pain in her left leg for six months. The patient was known to be hypertensive and on regular antihypertensive medication. She has smoked bidis for the last 25 years. No similar past or familial history was present. General examination revealed the presence of pallor and bipedal edema, while local examination indicated mild swelling on her left calf, which was non-tender upon palpation.

Hematological parameters were within normal limits, except for a reduced hemoglobin level (Table 1).

**Table 1:** Hematological parameters

S. No.	Parameters	Patient’s value	Reference range
1	White blood cell (WBC)	4.6 (1,000/μl)	4-11 (1,000/μl)
2	Red blood cell (RBC)	3.2 (1,000,000/μl)	4.5-5.5 (1,000,000/μl)
3	Hemoglobin	7.5 (g/dl)	12-15 (g/dl)
4	Hematocrit	26.8 (%)	40-50 (%)
5	Mean corpuscular volume (MCV)	77.8 (fl)	80-96 (fl)
6	Mean corpuscular hemoglobin (MCH)	26.8 (pg)	27-32 (pg)
7	Mean corpuscular hemoglobin concentration (MCHC)	30.7 (g/dl)	32-36 (g/dl)
8	Platelet	250 (1,000/μl)	150-400 (1,000/μl)
9	Red cell distribution width-standard deviation (RDW-SD)	44.5 (fl)	38-46 (fl)
10	Red cell distribution width-coefficient of variation (RDW-CV)	13.3 (%)	11.6-14 (%)
11	Neutrophil	65.2 (%)	40-80 (%)
12	Lymphocyte	25.5 (%)	20-40 (%)
13	Monocyte	6.4 (%)	2-10 (%)
14	Eosinophil	2.9 (%)	1-6 (%)
15	Basophil	0 (%)	0-1 (%)

**Figure 1:**
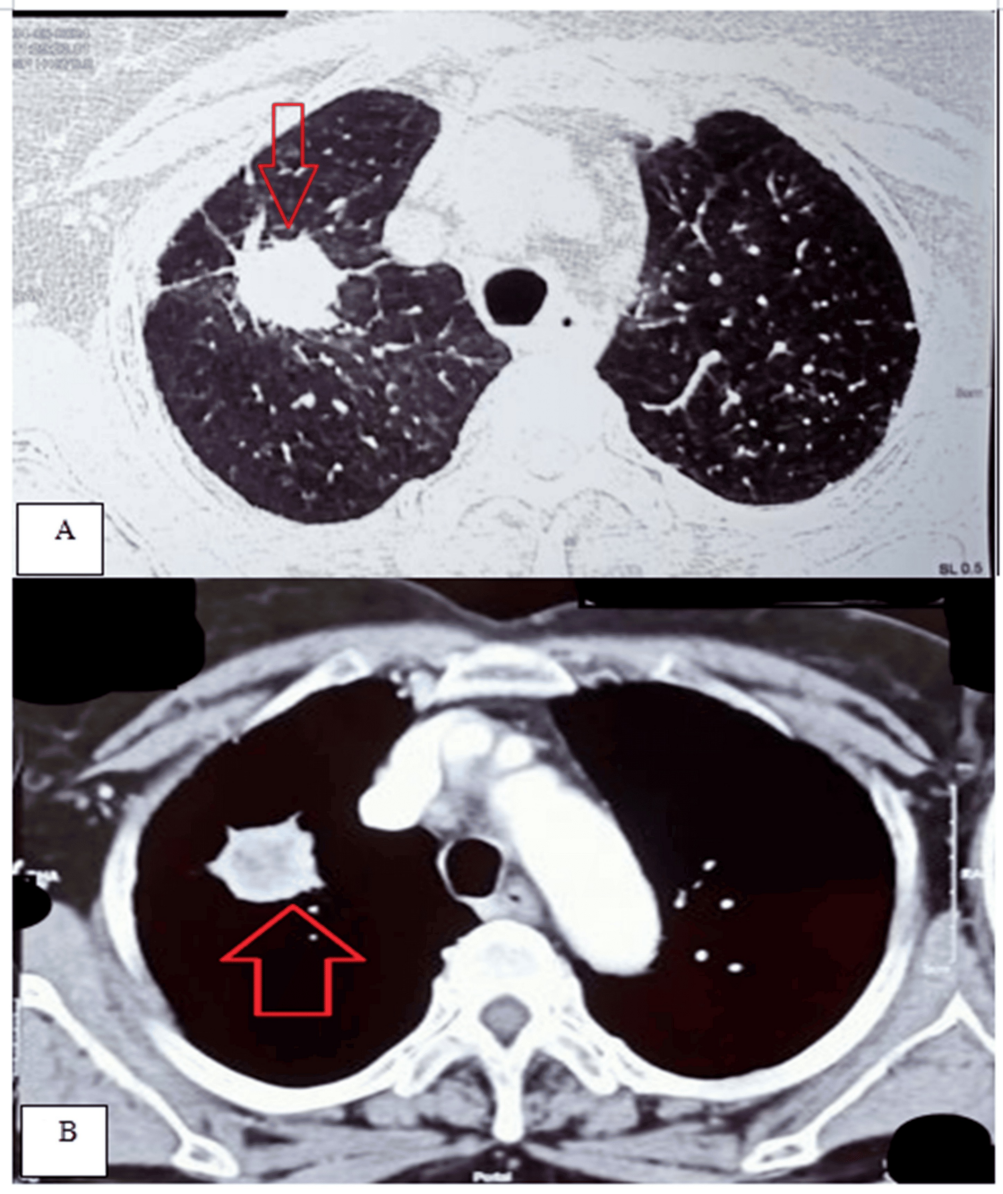
A) CT thorax (axial slice) showing soft tissue density lesion in the right upper lobe of the lung with heterogeneous enhancement (marked with a red arrow). B) CECT thorax (axial slice) showing irregular soft tissue density lesion in the right upper lobe of the lung with spiculated margins (marked with a red arrow)

Computed tomography (CT) scan of the thorax revealed a soft tissue density lesion in the right upper lobe of the lung with a heterogenous enhancement of 30 x 25 x 24 mm (Figure 1A), while a contrast-enhanced computed tomography (CECT) scan of the thorax showed a lesion with irregular and spiculated margins. Multiple prominent enlarged pretracheal and right paratracheal lymph nodes were also noted (Figure 1B). The above features suggested a bronchogenic malignancy with nodal metastases.

**Figure 2:**
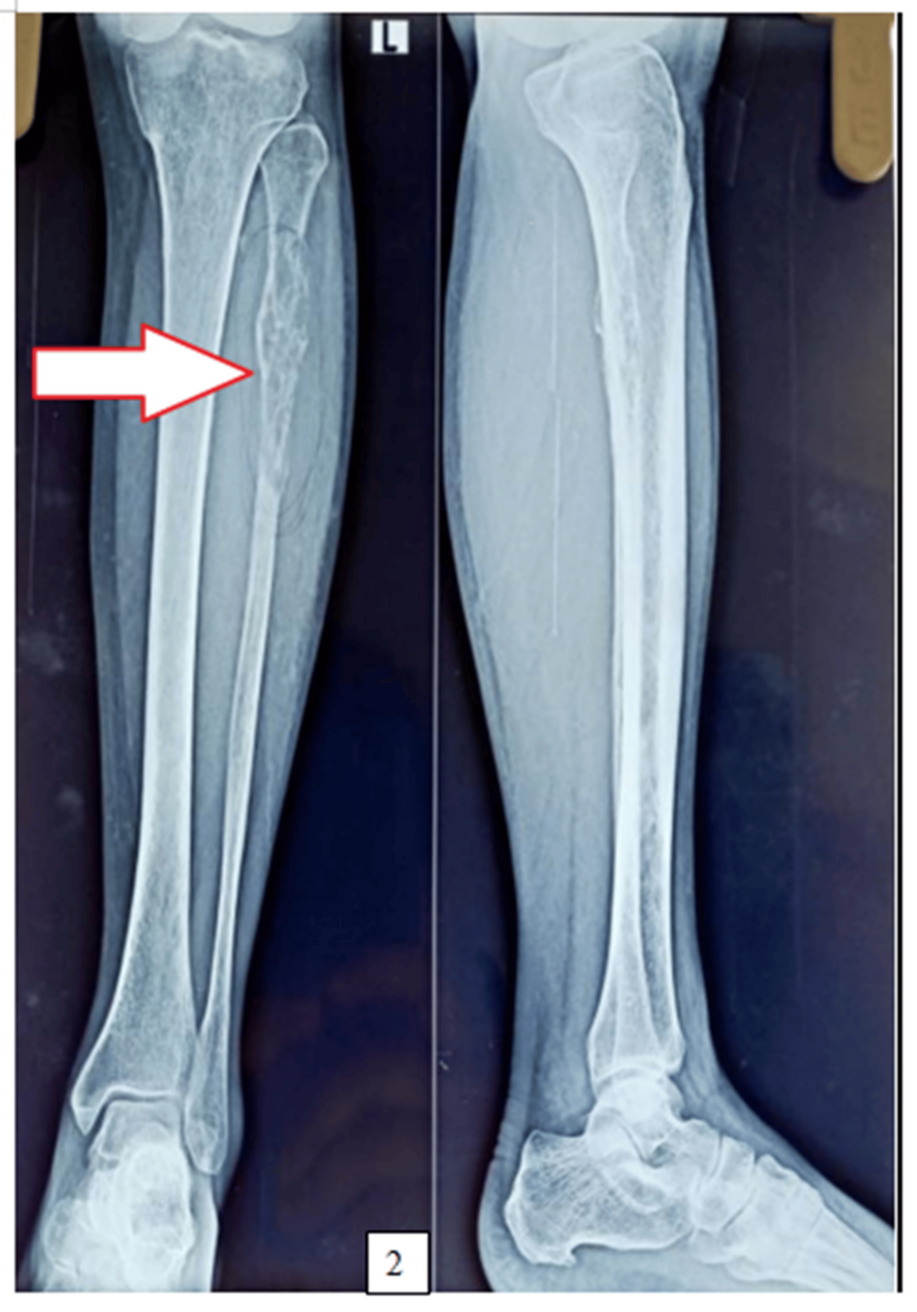
Radiograph: X-ray anteroposterior and lateral view of the left leg shows an expansile lytic lesion involving the proximal shaft of the fibula (marked with a white arrow)

The X-ray of the left lower leg revealed an osteolytic lesion (Figure 2). Contrast-enhanced magnetic resonance imaging (CEMRI) of the left lower limb also showed a malignant expansile lytic lesion with a soft tissue component involving the dia-metaphyseal region of the left proximal fibula, which was more likely indicative of osteosarcoma than any other diagnosis.

Moreover, a bone scan indicated increased radiotracer uptake in the left proximal fibula, which was consistent with left fibular osteosarcoma. Nonetheless, there was no abnormal tracer uptake in the rest of the axial and appendicular skeleton. In addition, a biopsy of the left fibular lesion done outside was negative for malignancy.

**Figure 3:**
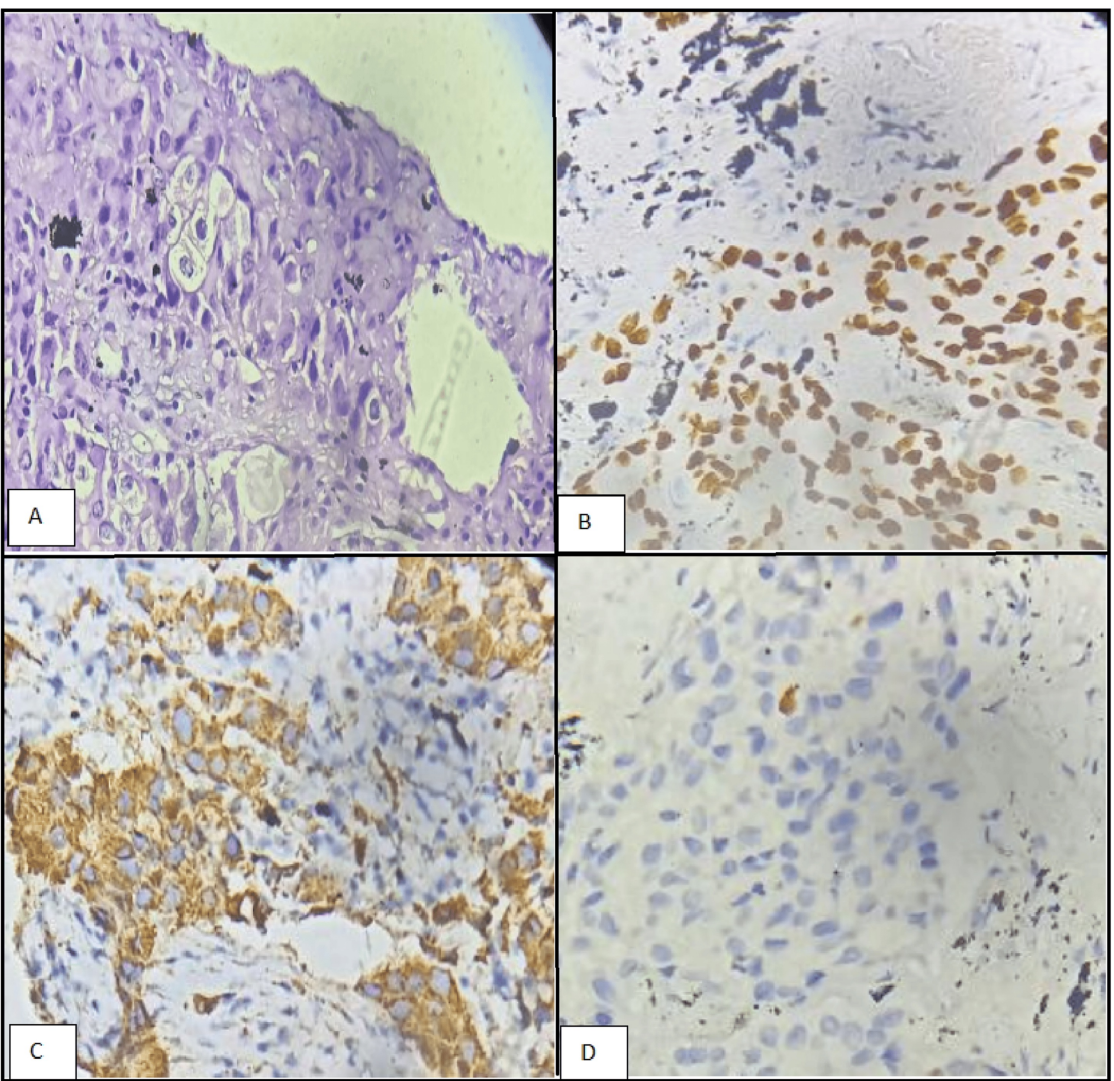
Biopsy from the lung showing adenocarcinoma A) Hematoxylin and eosin (H & E) (400X) shows a tumor arranged in nests and compact glands. B) IHC for TTF1 (400X) shows strong nuclear positivity in the tumor cells. C) IHC for Napsin A (400X) shows strong cytoplasmic granular positivity in the tumor cells. D) IHC for P40 (400X) shows no staining, ruling out squamous cell carcinoma

**Figure 4:**
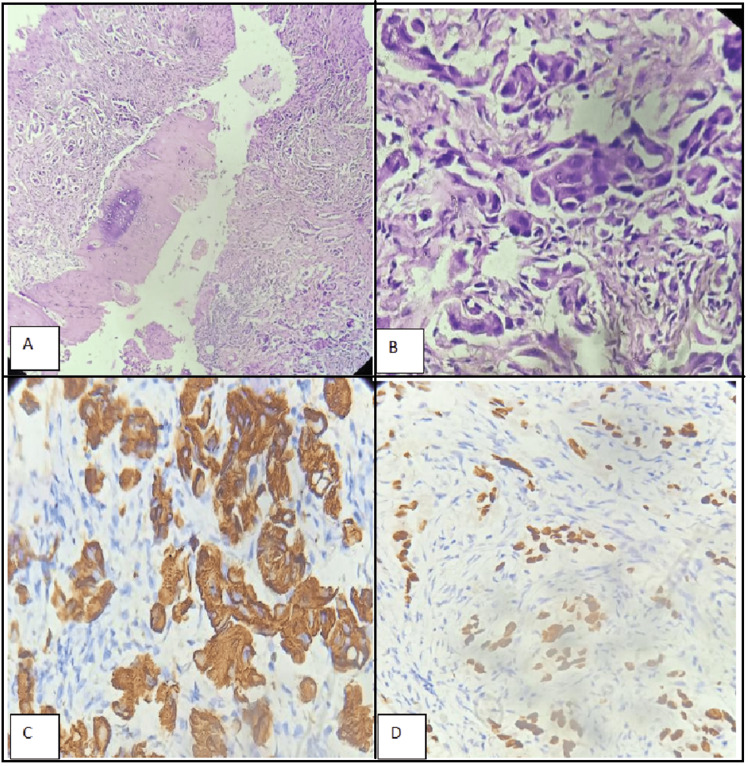
Biopsy from the left fibula showing metastatic lesion in a known case of lung adenocarcinoma A) H & E (40X) shows a tumor in nests and glands infiltrating into the bony trabeculae. B) H & E (400X) shows malignant cells with nuclear pleomorphism. C) IHC for CK7 (400X) shows strong membranous and cytoplasmic positivity in the tumor cells. D) IHC for TTF1 (400X) shows strong nuclear positivity in the tumor cells

The patient came to our institute for further treatment of double primary malignancy. A biopsy of her right lung mass showed lung adenocarcinoma (Figures 3A-3D); however, her left fibular lesion, which was biopsied with a clinical suspicion of osteosarcoma, showed tumor morphology similar to that of the lung biopsy (Figures 4A-4B). Immunohistochemistry (IHC) revealed cytokeratin (CK) 7 (Figure 4C) and thyroid transcription factor (TTF) 1 (Figure 4D) positivity, further corroborating metastasis from the lung primary. Molecular studies showed epidermal growth factor receptor (EGFR) positivity; however, anaplastic lymphoma kinase (ALK), reactive oxygen species (ROS), and phosphatidylinositol-4,5-bisphosphate 3 kinase catalytic domain (PIK3CA) were negative, making her a suitable candidate for EGFR-targeted tyrosine kinase inhibitor therapy.

After being evaluated in medical oncology, the patient was then referred to the pain and palliative care department. She took 250 mg of gefitinib tablets daily for primary lung adenocarcinoma and received monthly injections of zoledronic acid for three months to alleviate bone pain. If her bone pain worsened, she would have been eligible for palliative radiotherapy for her fibular metastasis. Nevertheless, she was doing well after three months of follow-up.

## Discussion

The most frequent metastatic sites of primary lung carcinoma are the nervous system, bone, liver, respiratory system, and adrenal gland. Liver and nervous system metastases are common in small-cell lung cancer patients, while bone and respiratory system metastases are prevalent in those with lung adenocarcinoma [4]. Milovanovic et al. found that among 175 patients, those with lung cancer metastases were over 50 years old and had adenocarcinoma as the most frequent histological type.

Metastases to adrenal glands are typically caused by adenocarcinoma and large-cell carcinoma, whereas those to the intestines are from large-cell carcinoma [5]. Metastases to bone are prevalent in many malignancies [2], with most occurring in the axial rather than the appendicular skeleton [6]. Within the appendicular skeleton, acrometastases are even rarer, accounting for only 0.1% of all bony metastases [7].

Lung carcinoma is the most common malignancy with acral metastases, followed by renal-cell carcinoma, breast carcinoma, and colon carcinoma. Lung carcinomas peak with acrometastases due to their direct access to the systemic arterial circulation, unlike other types of carcinoma that must pass through capillary channels in the lungs and liver before metastases can occur [8].

Adenocarcinoma of the lung is the most prevalent malignancy among the various lung carcinoma subtypes that present with both bone and acral metastases [9]. Among 2,021 lung cancer patients, Zhou et al. found that 23.9% had bone metastasis, with adenocarcinoma being the predominant histologic type to metastasize [10]. Radeczky et al. also identified that among 209 patients with metastatic adenocarcinoma of the lung to bone, the frequency of bony metastases at different sites, in descending order, was: spine (103), ribs (60), pelvis (36), femur (22), humerus (17), skull (13), sternum (10), clavicle (10), and scapula (10), with no instances of metastasis to acral sites, indicating their extremely rare occurrence [11].

Osteosarcoma, a differential diagnosis in our case, is a bone malignancy in the young, primarily affecting the distal femur, proximal tibia, and proximal humerus [12]; only 2% of cases occur in the proximal fibula [13].

The treatment of acral metastasis depends on the stage and extent of the tumor, involving amputation when feasible or palliative measures such as radiotherapy, bisphosphonates, and chemotherapy [8]. Table 2 shows several cases of bony acral metastases.

**Table 2:** Various cases of bony acral metastasis

S. No.	Case study	Year	Sex	Age (years)	Primary tumor	Site of acral metastasis
1	Soylemez et al. [14]	2015	Male	46	Squamous-cell carcinoma of the lung	Right hand - middle phalanx of index finger; left hand - distal phalanx of little finger
2	Dasan et al. [15]	2015	Male	62	Lung adenocarcinoma	Left fibula
3	Akram et al. [16]	2017	Male	50	Squamous-cell carcinoma of the lung	Right fibula
4	Tan and Lateef [3]	2020	Not mentioned	Elderly	Renal-cell carcinoma	Right middle finger, left ring finger
5	Mohammad and Ramakrishnan [17]	2024	Female	60	Lung adenocarcinoma	Left fibula
6	Fones et al. [18]	2024	Female	66	Squamous-cell carcinoma of the lung	Left middle and ring finger metacarpals
7	Present case	2024	Female	60	Lung adenocarcinoma	Left fibula

## Conclusions

In any case of a malignant solid tumor, it is critical to consider metastasis, especially when a suspicious focus is evident on radiological imaging. The incidence of multiple primary malignancies is increasing with time. Hence, although it is rare, the possibility of acral metastasis should always be taken into account when evaluating a case of a malignant solid tumor presenting with a swelling or a lytic lesion in the distal sites of the appendicular skeleton.
